# Prevalence and Progression of Resting ACTH, Insulin and Adiponectin Values as Indicators of Suspected Endocrine Diseases in Sport Horses and Ponies Compared to Non-Sport Horses, Ponies and Donkeys

**DOI:** 10.3390/ani15091316

**Published:** 2025-05-01

**Authors:** Emma Louise Davis, Andrew Douglas Wood, Julie F. N. Potier

**Affiliations:** 1 Rossdales Equine Hospital and Diagnostics Centre, Cotton End Road, Exning, Newmarket CB8 7NN, Suffolk, UK; 2 Liphook Equine Hospital, Forest Mere, Liphook GU30 7JG, Hampshire, UK

**Keywords:** equine, endocrine, sport medicine, PPID, metabolic obesity

## Abstract

Prevalence of endocrine disease in sport horses has been sparsely reported. They often compete well into their teenage years; thus, diagnosis and control of endocrine disease could be important to maintain performance and optimise health and welfare. The aim was to compare the prevalence of pituitary pars intermedia dysfunction (PPID), insulin dysregulation (ID) and metabolic obesity between sport and non-sport type breeds and assess disease progression. Blood samples submitted for plasma adrenocorticotropic hormone (ACTH), serum insulin or adiponectin were included in this study. Horses were grouped according to their breed into sport horses, which are typically used for athletic purposes, and non-sport horses, which are less-athletic breeds typically used for leisure. Cases included 1365 non-sport horse initial samples with 1850 non-sport horse follow-up samples and 1022 sport horse initial samples with 1081 sport horse follow-up samples. PPID and metabolic obesity were found to be as prevalent in the sport horse population, but non-sport horses were more likely to have ID. Upon follow up examination PPID was more prevalent with higher median ACTH in sport horses. This was also true of metabolic obesity in sport horses at follow-up, although median adiponectin was higher, perhaps indicating that the population is less severely affected than non-sport horses. Screening for endocrinopathies should be considered in sport horses presenting with poor performance.

## Simple Summary

Endocrine disease is commonly seen in aging horses and can be associated with clinical signs from poor performance to laminitis; however, the prevalence of endocrine disease in sport horses has been sparsely reported. Horses used for sports such as eventing, dressage and showjumping often compete into their teenage years and therefore, the diagnosis and control of endocrine disease could be important to optimise health and welfare. The aim of the study was determining how widespread endocrine conditions are within Sport horse breeds used for athletic purposes compared to non-sport horse breeds generally used for leisure. Blood samples submitted for endocrine testing were included in the study. An analysis of 3935 non-sport horses and 2113 sport horse samples showed some endocrine conditions were equally widespread between the two populations, but others were more prevalent in the sport horse population. Screening for endocrine disease should be considered in mature Sport horses and could enhance the future health and welfare of older equine athletes.

## 1. Introduction

Endocrine diseases in horses such as equine metabolic syndrome (EMS) and pituitary pars intermedia dysfunction (PPID) have been associated with increased age [1,2], with one in five horses aged 15 or older being at risk of PPID [3,4]. Evidence also suggests that between 22% and 27% horses are at risk of insulin dysregulation (ID) and that this risk increases with age [5,6,7]. Breed predispositions have been suggested to influence PPID [8,9] and ID status, with native breeds and ponies being more at risk [10,11]. Warmbloods such as Finnhorses [12] and other performance horses such as Arabians [13,14] have been shown to be genetically susceptible to equine metabolic syndrome (EMS). Insulin dysregulation is a feature of horses affected by EMS [15], can also be detected in horses with PPID [16] and is the primary cause of endocrinopathic laminitis [17,18].

Sport horse breeds are defined as horses or ponies typically bred for the purpose of performing in the disciplines of dressage, showjumping, eventing, western riding, driving or vaulting, while non-sport horses include horse and pony breeds of more native or cold-blooded phenotypes. In most athletic disciplines, sport horses reach their primes when they are around 10 years old, with many continuing to compete and perform into their late teenage years: for example, during the 2024 Paris Olympic games, only six out of 176 finishers in showjumping, dressage and eventing were under the age of 10, with 36 horses being older than 15 years of age [19]. Although not commonly reported, sport horses can be affected by endocrine diseases, which could be detrimental to their performances.

In addition to these risk factors, equine athletes are more likely to be treated with intra-articular corticosteroids [20] and are often fed sugar and starch-rich diets as primary sources of energy [21,22], which can further contribute to ID [23]. Furthermore, PPID has been linked to poor performance, with clinical signs such as lethargy and muscle wastage commonly reported [24]. Horses with PPID are also possibly at higher risk of tendon and ligament injury [25,26] and have been shown to be more likely to present with suspensory ligament degeneration [27].

Treatments for the aforementioned endocrine conditions include the dopamine agonist pergolide mesylate, which is licensed for PPID. This medication is commonly used and has a positive impact on the quality of life of horses with PPID [28]. In addition to pergolide, other off-license medications used for the treatment of ID, such as the antihyperglycaemic sodium–glucose transporter inhibitors canagliflozin or metformin, are currently controlled medications under most competition governing bodies (Fédération Equestre Internationale [29]), restricting their use in equine athletes. Thus, the need arises to assess the endocrine status of sport horses and the progression of the related blood markers in comparison with non-sport horses.

The aim of this study was to utilise a large database to investigate the initial samples submitted for diagnosis and the follow-up samples submitted for the monitoring of the plasma adrenocorticotropic hormone (ACTH), serum insulin and adiponectin in sport horses and ponies in comparison with non-sport breeds, to assess the progression of endocrine disease in these populations. Our hypothesis was that endocrine diseases such as metabolic obesity, insulin dysregulation and PPID would be as prevalent in sport horses as in non-sport horse breeds and that the disease progression would be similar in all cases, with an overall improvement of the endocrine analytes at follow-up compared with the initial samples.

## 2. Materials and Methods

### 2.1. Data Examined

Laboratory submissions were examined from the Liphook Equine Hospital between January 2020 and January 2024. Cases were included if blood samples had been submitted on the basis of clinical suspicion of endocrine diseases for measurements of plasma ACTH, serum insulin or adiponectin, or a combination of these endocrine markers. Laboratory submission regulations state that samples for ACTH should be chilled within 3 h of collection into EDTA blood tubes, and samples for insulin and adiponectin should be collected into serum blood tubes and sent to the laboratory via overnight delivery in custom-designed packaging to maintain a temperature of <4 °C. Analysis of the samples was performed within 4 h of receipt using the Immulite 2000 XPi Immunoassay (Siemens Healthcare Limited, Camberly, UK) using chemiluminescent methodology, and chilling was maintained up to the point of analysis.

Cases were excluded if the sample was not received in accordance with the submission protocol, if the submission form presented with no age or breed or if no clinical history was available. The first test samples included blood submitted for an initial diagnosis, as stated in the clinical history, and the follow-up samples comprised blood for which the clinical history clearly stated that it was a repeat sample. The sport horse group included samples from breeds such as Warmbloods, Thoroughbreds, Irish Sport Horses, Spanish and Arabians, which are typically used in common athletic riding disciplines (jumping, dressage, eventing, driving, endurance, vaulting, western) (Table 1). The non-sport horse group included equids typically not used for athletic purposes, which included native ponies, heavy draught horses, Cobs, miniatures horses and donkeys. The individuals were classified into the following categories according to their basal blood results, according to the recommendations for diagnosis and management of PPID and EMS guidelines for animals with low clinical suspicion [30,31]:

- PPID likely, or positive cases in which the basal ACTH was found to be above the upper end of the grey zone of each specific weekly reference interval previously published (95% specific for the diagnosis of PPID) [32];
- PPID unlikely, or negative cases in which basal ACTH was found to be below the lower end of the grey zone of the weekly reference intervals (95% sensitive for the exclusion of PPID diagnosis);
- ID likely, or positive cases in which the resting insulin was found to be higher than the 45 mIU/L cutoff adapted from the previous literature [33,34] and conversion webapp [35];
- ID unlikely, or negative cases when the resting insulin was below 32 mIU/L;
- Metabolically obese likely, or positive cases in which the adiponectin concentration was found to be lower than 7.9 mIU/L [36] (animals at a higher risk of developing insulin dysregulation and laminitis);
- Metabolically obese unlikely, or negative animals in which adiponectin concentrations were above 8 mIU/L.

### 2.2. Statistical Analysis

All analyses were performed using R Statistical Software (v4.1.2; R Core Team 2021) [37]. Contingency tables were constructed according to the endocrine disease status (positive or negative), and χ^2^ and Fisher’s exact tests were used to compare the frequencies. The graphical distribution of the data and quantile–quantile plots were assessed for normality. Normally distributed data were compared using a paired Student’s test, and data not normally distributed were compared using a Wilcoxon signed-rank test. Statistical significance was set at *p* ≤ 0.05.

## 3. Results

### 3.1. Demographics

Initially, 241,815 laboratory submission records were examined, and following application of the exclusion criteria, 3935 non-sport horse and 2113 sport horse cases were selected (Table 1). Among the non-sport horse samples, 1365 were initial tests, with 1850 being follow-up samples. Similarly, among the sport horse samples, 1022 were initial tests, while 1081 were follow-up samples.

**Table 1:** Breed demographics in the sport horse and non-sport horse groups.

Sport Horses		Non-Sport Horses	
Warmblood types	714	Native ponies	1976
Baroque type sport horses	68	Heavy Draughts	14
Arabian horses	142	Cobs	675
American sport horses	57	Miniatures	23
Sport ponies	187	Heavy ponies	44
Racehorses	312	Donkeys	94
Cross breeds	633 crossed from above	Cross breeds	1109 crossed from above
Total horses	2113	Total horses	3935

In the non-sport horses, the population age ranged from 0 to 44 years, with a median age of 20 years old (IQR 16–25). The data included samples from 1698 (43%) mares, 252 (6%) stallions and 1874 (48%) geldings, while the sex was not mentioned in 111 cases.

In the sport horses, the population age ranged from 0 to 37 years, with a median age of 21 years old (IQR 17–25). The data included samples from 803 (38%) mares, 138 (7%) stallions and 1135 (54%) geldings, while the sex was not mentioned in 37 cases.

Median age and sex distribution were not significantly different between the non-sport horses and the sport horses.

### 3.2. Submission Data

**Figure 1:**
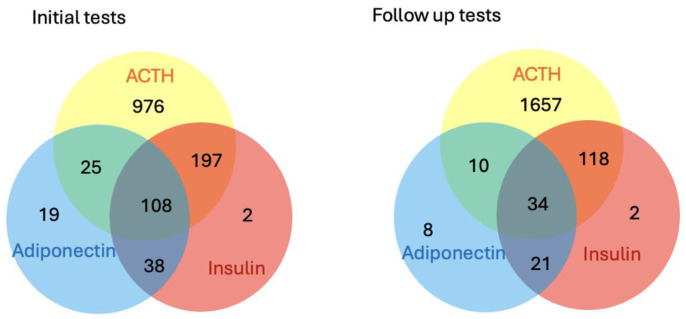
Description of the tests submitted for non-sport horses from the initial (n = 1365) and follow-up (n = 1850) samples.

For the initial diagnostic tests on the non-sport horses, there was a total of 1306 submissions for ACTH, 345 for insulin and 190 for adiponectin. Of these, 976 were submitted for ACTH only, 2 for insulin only, 19 for adiponectin only, 38 for insulin and adiponectin, 197 for insulin and ACTH, 25 for ACTH and adiponectin and 108 for ACTH, insulin and adiponectin. For the follow-up samples, there was a total of 1819 submissions for ACTH, 175 for insulin and 73 for adiponectin. Of these, 1657 were submitted for ACTH only, 2 for insulin only, 8 for adiponectin only, 21 for insulin and adiponectin, 118 for insulin and ACTH, 10 for ACTH and adiponectin and 34 for ACTH, insulin and adiponectin (Figure 1).

**Figure 2:**
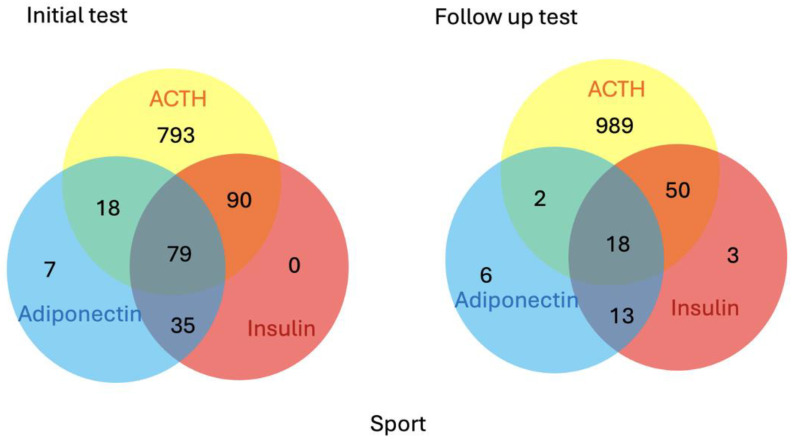
Description of the tests submitted for sports horses from the initial (n = 1022) and follow-up (n = 1081) samples.

For the initial diagnostic tests on the sport horses, there was a total of 980 submissions for ACTH, 204 for insulin and 139 for adiponectin. Of these, 793 were submitted for ACTH only, 7 for adiponectin only, 35 for insulin and adiponectin, 90 for insulin and ACTH, 18 for ACTH and adiponectin and 79 for ACTH, insulin and adiponectin. For the follow-up samples, there was a total of 1059 submissions for ACTH, 84 for insulin and 39 for adiponectin. Of these, 989 were submitted for ACTH only, 3 for insulin only, 6 for adiponectin only, 13 for insulin and adiponectin, 50 for insulin and ACTH, 2 for ACTH and adiponectin and 18 for ACTH, insulin and adiponectin (Figure 2).

### 3.3. Endocrine Status

The majority of the samples (96%) included in the final analysis were obtained between November and May; therefore, seasonal variations of the analytes were minimal. The classification of the cases according to their suspected endocrine disease statuses is summarized in Table 2. There was no difference in the frequency of PPID or metabolic obesity in the initial samples between the sport horses and the non-sport horses. The sport horse population was significantly more likely to have PPID cases at the follow-up sample (567/1059, 53.1%) compared with the non-sport horses (854/1819, 46.5%) (*p* < 0.001). The sport horse population was significantly less likely to have insulin-dysregulated cases than the non-sport horse population for both the initial (68/204, 33.3% and 173/345, 50.1%, respectively) (*p* < 0.001) and follow-up samples (31/84, 36.9% and 105/175, 60%, respectively) (*p* < 0.001). Conversely, the sport horse population presented with more suspected metabolically obese individuals at follow-up (17/39, 43.6%) than the non-sport horses (16/73, 21.9%) (*p* = 0.03).

### 3.4. Analytes Analysis

**Figure 3:**
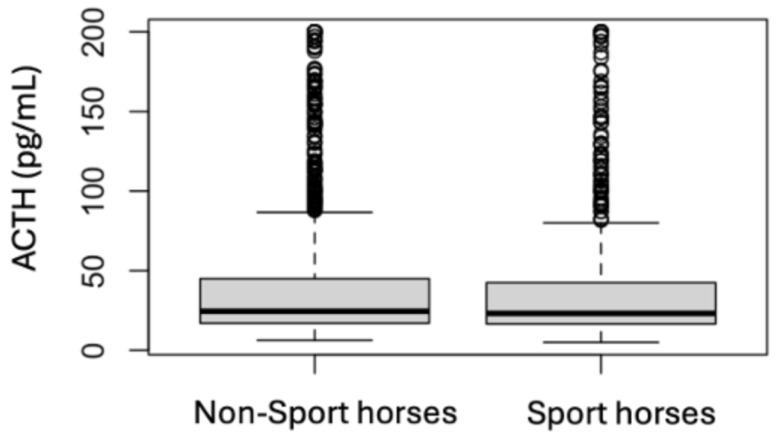
Comparison of the initial ACTH results for non-sport horses and sport horses (respectively 94 and 45 outliers with results > 200 pg/mL).

**Figure 4:**
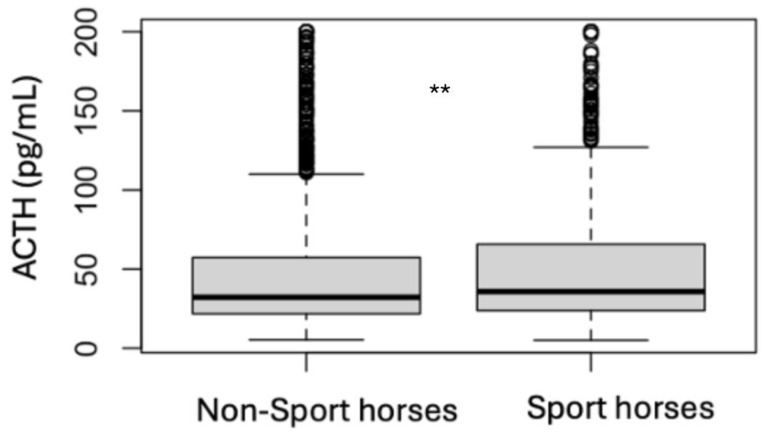
Comparison of the follow-up ACTH results for non-sport horses and sport horses (** *p* < 0.01) (respectively 103 and 66 outliers with results > 200 pg/mL).

**Figure 5:**
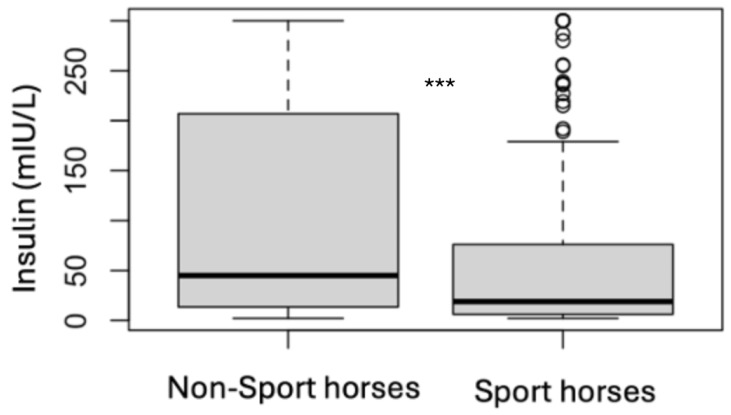
Comparison of the initial insulin results for non-sport horses and sport horses (*** *p* < 0.001).

**Figure 6:**
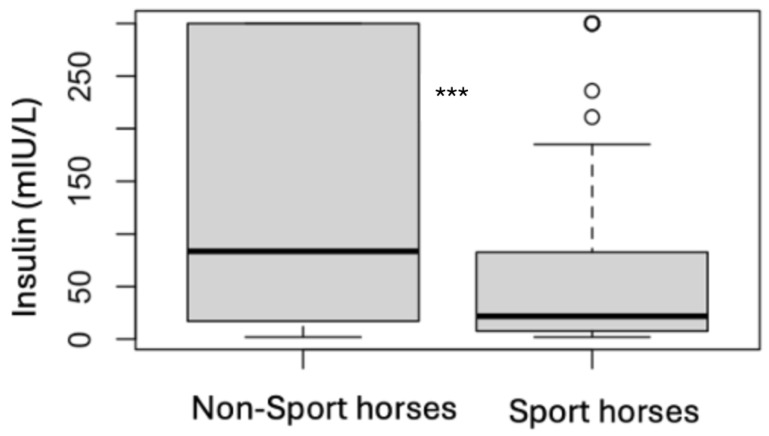
Comparison of the follow-up insulin results for non-sport horses and sport horses (*** *p* < 0.001).

**Figure 7:**
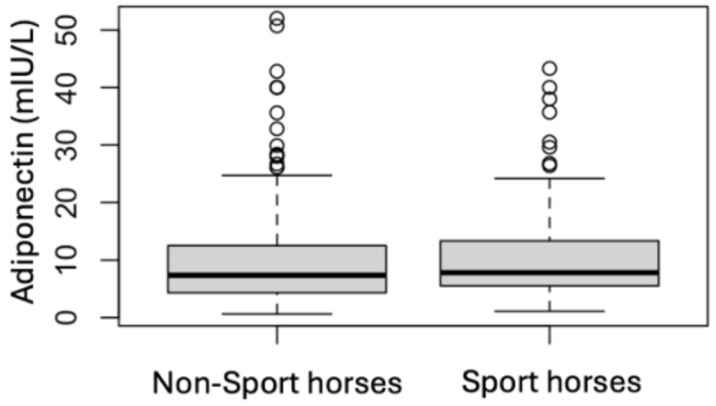
Comparison of the initial adiponectin results for non-sport horses and sport horses.

**Figure 8:**
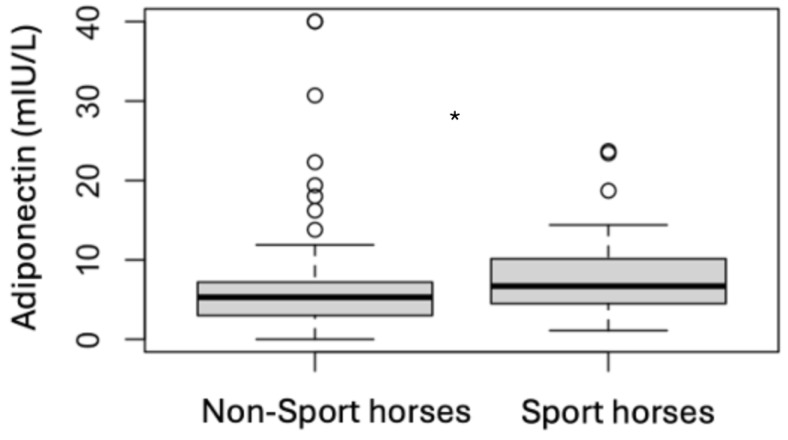
Comparison of the follow-up adiponectin results for non-sport horses and sport horses (* *p* < 0.05).

Median values and interquartile ranges for each analyte are presented in Table 3 for the initial samples and in Table 4 for the follow-up samples. The median initial ACTH for the non-sport horses (24.5 pg/mL) was not different than the median initial ACTH for the sport horses (23.5 pg/mL) (Figure 3), but when the follow-up samples were examined, the median ACTH for the non-sport horses (32.2 pg/mL) was lower than the median ACTH for the sport horses (35.8 pg/mL) (*p* = 0.002) (Figure 4). The median ACTH at follow-up was higher than the initial ACTH for the non-sport horses and the sport horses (*p* < 0.001 for both). The median initial insulin was higher for the non-sport horses (44.9 mIU/L) than for the sport horses (18.9 mIU/L) (*p* < 0.001) (Figure 5). The follow-up samples showed the same difference, with the median follow-up insulin for the non-sport horses (83.5 mIU/L) being higher than the median follow-up insulin for the sport horses (21.8 mIU/L) (*p* > 0.001) (Figure 6). There was no difference between the median initial and the follow-up insulin for the sport horses, while the median initial insulin was lower than the follow-up insulin for the non-sport horses (*p* = 0.03). The median initial adiponectin for the non-sport horses (7.4 mIU/L) was not different than the median initial adiponectin for the sport horses (7.8 mIU/L) (Figure 7), but the median follow-up adiponectin for the non-sport horses (5.3 mIU/L) was lower than the median follow-up adiponectin for the sport horses (6.7 mIU/L) (*p* = 0.04) (Figure 8). The median adiponectin at follow-up was lower than the initial adiponectin for the non-sport horses (*p* < 0.001) and for the sport horses (*p* = 0.04)

## 4. Discussion

The results of this study indicate that endocrine disorders are likely common in competition and performance horses, with suspected PPID and metabolic obesity being found to have a similar incidence in the sport horse population as the non-sport horse population. Additionally, an increased likely prevalence at the follow-up sampling was observed for both diseases in both groups. Possible insulin dysregulation appeared to be more prevalent in the non-sport horse population, with more positives and higher median insulin results in both the initial and follow-up samples.

Contrary to the hypothesis, the suspected endocrine diseases progressed at follow-up. PPID especially seemed to be likely to be more prevalent at follow-up examinations in the sport horses than in the non-sport horses, with a higher median ACTH for the sport horses’ follow-up samples compared with the non-sport horses. A possible logical explanation is that ACTH tends to increase with age [38], and the follow-up samples are by definition from older horses than the initial test ones. This explains the upward trend of the median ACTH, but not the difference between the sport horses and the non-sport horses, as the same bias would apply for both groups. A more concerning explanation for the difference between the sport horses and the non-sport horses would be that horses diagnosed with PPID and still actively competing are not placed on pergolide mesylate treatment due to the concern regarding medication and doping control from competition governing bodies and therefore have uncontrolled PPID. This is a potential welfare issue in sport horses, as not only have endocrine diseases such as PPID and EMS been associated with poor performance [26], they have also been associated with laminitis [17,39], which could be career-ending or even fatal when the condition is advanced [40,41]. A possible nuance to this explanation is that there is also an increase in median ACTH in the non-sport horse population, although to a lesser extent.

Endocrine disorders are commonly associated with joint and tendon injury in humans [42,43]. Similarly, PPID has been linked with suspensory ligament degeneration in horses, with the supposed pathophysiological explanation that high levels of glucocorticoids are thought to reduce the tensile strength of the ligament, hence playing a role in degeneration [27]. Suspensory desmitis is a common disease of the sport horse [44] and can significantly impact the careers of equine athletes. There is also evidence suggesting a potential increase in suspensory desmopathy in sport horses over 10 years old showing signs of PPID [25]. Given the impact on performance and the high incidence of suspensory desmopathy within the sport horse population, the early detection and treatment of PPID could help contribute to a decreased occurrence of suspensory desmopathy and degeneration.

The metabolic obesity status is interesting, as there were more cases of suspected metabolic obesity in the sport horse cases at follow-up. This is concerning, as obesity itself can lead to poor performance and has been linked with lameness exacerbation [25,45]. In addition, nasopharyngeal collapse has been anecdotally reported in obese horses [46]. In humans, this is postulated to be due to the secretion of adipokines, which can disturb the neuromuscular control of the pharynx and significantly impact performance [47]. Obesity in horses can also contribute to insulin dysregulation and therefore increase the risk of performance-limiting laminitis [26]. Interestingly, the median adiponectin was found to be lower for the non-sport horse samples, meaning that metabolic obesity is potentially less frequent but perhaps more severe in non-sport horses. This is likely due to some native breeds such as Draught types and Welsh being particularly predisposed to obesity [48].

ID is most commonly associated with PPID and/or metabolic obesity in equids, and the insulin results at follow-up were found to be higher than the initial samples for both the non-sport horses and the sport horses but were significantly higher for the non-sport horses. This could be due to sport breeds being used for athletic purposes and therefore, these individuals have more muscle mass and subsequent increased muscle-peripheral insulin sensitivity [49,50]. Different breeds of horses and ponies have been involved in corticosteroid-induced laminitis cases [20], which are usually associated with underlying endocrinopathies, especially insulin dysregulation. The use of corticosteroids in sport horse practice is commonplace, in particular for intra-articular medication [51], which is more likely to be required for the orthopaedic management of older sport horses [52]. Given the prevalence of endocrine diseases within older sport horse types, it would be prudent to rule out the presence of any endocrine disorder and especially insulin dysregulation, with appropriate testing [33], prior to use of corticosteroids [53].

The potential influence of underlying endocrine disease when assessing poor performance in equine athletes should therefore be considered. There are important welfare considerations regarding the treatment of endocrine disease, given that the medications recommended for treatment, such as pergolide mesylate, are often controlled under governing bodies [29]. However, the use of the controlled medication is often not feasible for competing horses because competition schedules may not allow for the adequate withdrawal of medication. There is also the added concern regarding the continued withdrawal and re-introduction of the medication about not only the effectiveness of treatment but also the potential detrimental effects of such use. In humans, it has been previously reported that the withdrawal of medications can cause rebound secretions in cases of pituitary adenomas [54,55,56], but there is no evidence as to the effect of treatment withdrawal on long-term management of endocrine conditions in horses. Given the difficulty surrounding the use of pergolide mesylate in competition horses, in some cases, it may result in the inability of teenage sport horses to continue competing due to clinical signs of uncontrolled PPID, which may result in early retirement (unpublished data). Further work is therefore required to ascertain the effect of a short withdrawal of these medications as well as to determine if there is any basis for their prohibition. No published scientific evidence of the performance-enhancing effects of these drugs currently support the need for them to be labelled as controlled medications.

The limitations of the current study include the data and samples being supplied by a multitude of veterinary surgeons, and while these are considered to be accurate, there was no verification of the data performed. The clinical data were submitted with each sample, but the history provided did not include the individual body condition scores or information regarding the competition status of the sport horse population and whether they were actively competing in athletic disciplines. It was not possible to obtain this information within the scope of this study. Although a protocol was given with regard to sampling handling, there was no means to verify that the advice was followed meticulously. The retrospective nature of the data is biased toward animals that were likely exhibiting clinical signs rather than randomised sampling of the general population; this could result in an overestimation of the prevalence of endocrinopathies in the general population. This being said, the same bias would be valid both for non-sport horses and sport horses; thus, the difference observed between the two populations should outweigh this bias. Another limitation is that the cutoffs used for diagnosing the diseases were highly specific cutoffs rather than grey zone or highly sensitive cutoffs: these cutoffs were selected because the completeness of description of the clinical signs displayed by each animal could not be ascertained, and therefore, a high specificity cutoff would allow the minimization of false negative results. This may underestimate the true prevalence of the disease in the populations, but once again, this bias should affect both the sport horse and the non-sport horse population equally. There are also possible biases regarding the diagnosis of PPID with regard to seasonality, although limited, and the possible interaction with the insulin dysregulation status of each individual. Horses were classified as ID-unlikely or negative when the insulin was below 32 mIU/L. Although this assumption is limited by the fact that the samples were resting and false negatives could have occurred, dynamic testing with oral carbohydrate challenge would optimise this category.

## 5. Conclusions

In conclusion, endocrine diseases, especially PPID and metabolic obesity, are suspected to be equally common in sport horse breeds compared to non-sport horse breeds. Follow-up samples in sport horses indicate a possible increase in these suspected endocrine disorders compared with non-sport horses. The clinical impact of these endocrinopathies warrants further investigation as to whether they could be a contributing factor in cases of poor performance. Future studies should be directed at assessing the possible link between endocrine diseases and factors affecting performance in equine athletes.

## Figures and Tables

**Table 2:** Numbers of cases and percentages classified by their suspected endocrine disease statuses (ID = insulin dysregulation. PPID positive cases had results above the weekly grey zone, while negative cases had results below the weekly grey zone, and ID positive cases had results above 45 mIU/L, while negative cases had results < 32 mIU/L; cases with results that fell into the grey zone were excluded from the likely diagnoses).

	Sport Horses		Non-Sport Horses	
	Initial	Follow-Up	Initial	Follow-Up
**PPID positive**	298 (30%)	567 (54%)	866 (66%)	854 (47%)
**PPID negative**	344 (35%)	138 (13%)	417 (32%)	323 (18%)
**ID positive**	68 (33%)	31 (37%)	173 (50%)	105 (60%)
**ID negative**	130 (64%)	48 (57%)	148 (43%)	60 (34%)
**Obesity positive**	73 (53%)	17 (44%)	86 (45%)	16 (22%)
**Obesity negative**	66 (47%)	22 (56%)	105 (55%)	57 (78%)

**Table 3:** Medians and interquartile ranges for the ACTH, insulin and adiponectin results of the initial and follow-up samples from non-sport horses (IQR = interquartile range).

	Initial Tests			Follow-Up Tests		
	ACTH (pg/mL)	Insulin (mIU/L)	Adiponectin (mIU/L)	ACTH (pg/mL)	Insulin (mIU/L)	Adiponectin (mIU/L)
**Median**	24.5	44.9	7.4	32.2	83.5	5.3
**IQR**	17.0–45.0	13.4–207.0	4.3–12.4	21.8–57.3	17.0–300.0	3.0–7.2

**Table 4:** Medians and interquartile ranges for the ACTH, insulin and adiponectin results of the initial and follow-up samples from the sport horses (IQR = interquartile range).

	Initial Tests			Follow-Up Tests		
	ACTH (pg/mL)	Insulin (mIU/L)	Adiponectin (mIU/L)	ACTH (pg/mL)	Insulin (mIU/L)	Adiponectin (mIU/L)
**Median**	23.2	18.9	7.8	35.8	21.8	6.7
**IQR**	16.6–42.4	6.2–74.7	5.5–13.4	23.8–65.7	7.8–80.4	4.5–10.2

## Data Availability

Data are available upon request due to restrictions.

## Abbreviations

The following abbreviations are used in this manuscript:

PPID	Pituitary pars intermedia dysfunction
ID	Insulin dysregulation
ACTH	Adrenocorticotropic hormone
EMS	Equine metabolic syndrome
